# Targeting the differential addiction to anti-apoptotic BCL-2 family for cancer therapy

**DOI:** 10.1038/ncomms16078

**Published:** 2017-07-17

**Authors:** Akane Inoue-Yamauchi, Paul S. Jeng, Kwanghee Kim, Hui-Chen Chen, Song Han, Yogesh Tengarai Ganesan, Kota Ishizawa, Sylvia Jebiwott, Yiyu Dong, Maria C. Pietanza, Matthew D. Hellmann, Mark G. Kris, James J. Hsieh, Emily H. Cheng

**Affiliations:** 1 Human Oncology and Pathogenesis Program, Memorial Sloan Kettering Cancer Center, 1275 York Avenue, New York, New York 10065, USA; 2 Department of Surgery, Memorial Sloan Kettering Cancer Center, New York, New York 10065, USA; 3 Thoracic Oncology Service, Department of Medicine, Memorial Sloan Kettering Cancer Center, New York, New York 10065, USA; 4 Department of Medicine, Weill Cornell Medical College, New York, New York 10065, USA; 5 Molecular Oncology, Department of Medicine, Siteman Cancer Center, Washington University, St Louis, Missouri 63110, USA; 6 Department of Pathology, Memorial Sloan Kettering Cancer Center, New York, New York 10065, USA; 7 Department of Pathology and Laboratory Medicine, Weill Cornell Medical College, Cornell University, New York, New York 10065, USA

## Abstract

BCL-2 family proteins are central regulators of mitochondrial apoptosis and validated anti-cancer targets. Using small cell lung cancer (SCLC) as a model, we demonstrated the presence of differential addiction of cancer cells to anti-apoptotic BCL-2, BCL-X_L_ or MCL-1, which correlated with the respective protein expression ratio. ABT-263 (navitoclax), a BCL-2/BCL-X_L_ inhibitor, prevented BCL-X_L_ from sequestering activator BH3-only molecules (BH3s) and BAX but not BAK. Consequently, ABT-263 failed to kill BCL-X_L_-addicted cells with low activator BH3s and BCL-X_L_ overabundance conferred resistance to ABT-263. High-throughput screening identified anthracyclines including doxorubicin and CDK9 inhibitors including dinaciclib that synergized with ABT-263 through downregulation of *MCL-1*. As doxorubicin and dinaciclib also reduced BCL-X_L_, the combinations of BCL-2 inhibitor ABT-199 (venetoclax) with doxorubicin or dinaciclib provided effective therapeutic strategies for SCLC. Altogether, our study highlights the need for mechanism-guided targeting of anti-apoptotic BCL-2 proteins to effectively activate the mitochondrial cell death programme to kill cancer cells.

## Abstract (web summary)

Small cell lung cancer cells (SCLC) are differentially sensitive to inhibitors of the BCL-2 family. Here the authors analyse the response to BH3 mimetics in SCLC, delineate patterns of expression of apoptotic proteins correlated with differential sensitivities and demonstrate a synergistic anti-tumour activity between ABT-199 and anthracyclines or CDK9 inhibitors.

The BCL-2 family proteins dictate cellular survival or death decisions by regulating the integrity of the mitochondrial outer membrane (MOM)^1^. Apoptotic signals culminate in MOM permeabilization (MOMP), prompting the release of cytochrome *c* and the activation of caspases. Initiation of the BCL-2 regulated cell death cascade occurs through the transcriptional and posttranslational activation of proapoptotic BH3-only molecules (BH3s), which serve as death sentinels that either directly activate multidomain proapoptotic BAX and BAK (‘activator’ BH3s) or inactivate multidomain anti-apoptotic BCL-2, BCL-X_L_ and MCL-1 (‘inactivator’ BH3s)^2^,^3^,^4^,^5^,^6^. BAX and BAK, the essential effectors of MOMP, undergo stepwise, bimodal conformational changes upon activation by the activator BH3s to form homo-oligomers that mediate cytochrome *c* efflux^4^,^6^,^7^. Conversely, anti-apoptotic BCL-2, BCL-X_L_ and MCL-1 preserve mitochondrial integrity through sequestration of activator BH3s or partially activated, BH3-exposed, BAX/BAK monomers to prevent the homo-oligomerization of BAX/BAK^1^,^2^,^3^,^4^,^6^,^8^. To evade apoptotic checkpoints, cancer cells often overexpress anti-apoptotic BCL-2 proteins^9^. Consequently, along with the fact that activation of the BCL-2-controlled apoptotic pathway seems critical for the efficacy of most chemotherapeutics, BCL-2 family members have emerged as attractive targets for therapeutic development.

Structure-based efforts led to the development of the first specific small molecule inhibitor of the BCL-2 family, ABT-737 and its orally bioavailable analog ABT-263 (navitoclax) that bind and inhibit BCL-2, BCL-X_L_ and BCL-W, but not MCL-1 or BCL2A1 (refs 10, 11, 12, 13, 14). Although navitoclax showed promising clinical activity, it induced a dose-dependent thrombocytopenia as an on-target result of BCL-X_L_ inhibition^15^,^16^. This spurred the development of ABT-199 (venetoclax or GDC-0199), a platelet-sparing, selective BCL-2 inhibitor^17^. Venetoclax has exhibited remarkable therapeutic efficacy for relapsed/refractory chronic lymphocytic leukaemia with an overall response rate of 79% (ref. 18), resulting in its approval by the Food and Drug Administration (FDA) for the treatment of chronic lymphocytic leukaemia patients with 17p deletion. Selective inhibitors for BCL-X_L_ with robust preclinical activity have also been generated^19^, but similar efforts to target MCL-1 have been less successful. The lack of effective MCL-1 inhibitors positions MCL-1 as a key primary as well as secondary resistance factor to ABT-263 and ABT-199.

Small cell lung cancer (SCLC) is an aggressive type of neuroendocrine carcinoma that represents 10–15% of all lung cancer malignancies^20^. Standard first-line treatment consists of a combined regimen of platinum-based chemotherapy with etoposide and typically elicits high initial response rates, followed by almost universal disease recurrence and progression^21^. As a result, 5-year survival rate is dismal (∼5%) with little improvement over the past 30 years^20^,^21^. Unlike non-SCLC, which is commonly associated with targetable kinase mutations, SCLC biology is less evidently tractable, driven instead by nearly uniform loss of tumour suppressors *TP53* and *RB1* (refs 22, 23). Preclinical studies showed that SCLC cell lines are among the most sensitive tumour types to ABT-737 and ABT-263 (refs 10, 11, 24, 25), suggesting that targeting the BCL-2 family proteins may be a paradigm shifting therapeutic strategy for this cancer. However, not all SCLC cell lines are sensitive to ABT-263 (refs 11, 24, 25) and limited single agent activity of navitoclax was observed in a phase II trial for SCLC^16^. It has become evident that combination therapy with ABT-263 is required to improve the therapeutic outcome of SCLC. However, it remains unclear how ABT-263 can be integrated with existing chemotherapeutics into rational combination treatments for SCLC, or if particular classes of targeted therapeutics will synergize favourably with ABT-263. Moreover, reliable biomarkers for identifying patient populations who will respond to ABT-263 monotherapy are yet unknown.

Using an unbiased high-throughput screening (HTS) strategy, we identified anthracyclines including doxorubicin and CDK9 inhibitors including dinaciclib that enhanced the proapoptotic effect of ABT-737/263 through downregulation of *MCL-1*. Both doxorubicin and dinaciclib also cooperated with ABT-199 to induce robust apoptosis, because they also partially reduced BCL-X_L_. The *in vivo* therapeutic efficacy of these combinations was demonstrated in mouse xenograft models, validating new potential therapeutic strategies for SCLC. Interestingly, we found that some SCLC cell lines displayed differential addiction to BCL-2, BCL-X_L_ or MCL-1 for survival, which could be determined by the respective protein expression ratio. Surprisingly, ABT-263 failed to kill BCL-X_L_-addicted cells with low expression of activator BH3s, as ABT-263 failed to prevent BCL-X_L_ from sequestering BAK in these cells. Consequently, overexpression of BCL-X_L_ conferred resistance to ABT-263, representing a previously unknown therapeutic limitation. Together, our data establish a predictive paradigm for determining SCLC addiction to anti-apoptotic BCL-2 family members and highlight the need for mechanism-guided targeting of anti-apoptotic BCL-2 proteins for effective apoptosis induction.

## Results

### HTS identifies anthracyclines that cooperate with ABT-263

**Figure 1:**
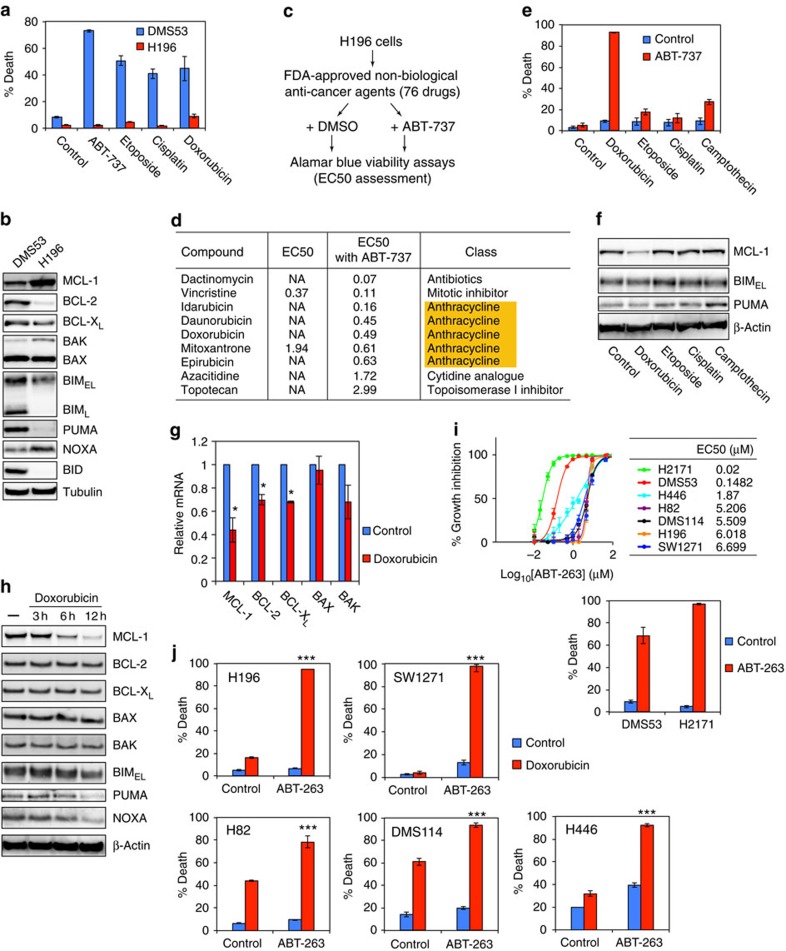
HTS of FDA-approved anti-cancer agents identifies anthracyclines that enhance the proapoptotic effect of ABT-737. (**a**) DMS53 and H196 cells were treated with the indicated agents for 48 h. Cell death was quantified by annexin-V staining (mean±s.d., *n*=3). (**b**) The expression of BCL-2 family proteins in DMS53 and H196 cells was assessed by immunoblot analysis. (**c**) A schematic of HTS to identify FDA-approved anti-cancer agents that cooperate with ABT-737 to reduce the survival of H196 cells. H196 cells were treated with each compound at 12 two-fold serial dilution doses starting from 10±1 μM ABT-737. Cell viability was assessed by Alamar Blue assays at 72 h and EC50 was calculated. (**d**) A summary of EC50s of anti-cancer agents±ABT-737 in H196 cells. NA denotes ‘not applicable’, because the agent failed to achieve 100% growth inhibition at 10 μM. (**e**) H196 cells were treated with the indicated agents for 48 h. Cell death was quantified by annexin-V staining (mean±s.d., *n*=3). (**f**) H196 treated with the indicated agents for 6 h were assessed by immunoblot analysis. (**g**) The mRNA levels of BCL-2 family in H196 cells treated with 2 μM doxorubicin for 3 h were assessed by qRT–PCR. Data were normalized against β-Actin (mean±s.d., *n*=2 independent experiments). (**h**) H196 cells treated with doxorubicin for the indicated times were assessed by immunoblot analysis. (**i**) A summary of EC50s of ABT-263 in SCLC cell lines. The indicated SCLC cell lines were treated with increasing concentrations of ABT-263. Cell viability was assessed by CellTiter-Glo assays at 48 h. DMS53 and H2171 cells were treated with 1 μM ABT-263 for 24 h and cell death was quantified by annexin-V staining (mean±s.d., *n*=3). (**j**) The indicated SCLC cell lines were treated with 2 μM doxorubicin±1 μM ABT-263 for 24 h. Cell death was quantified by annexin-V staining (mean±s.d., *n*=3). *P<0.05 and ***P<0.001 (*t*-test). Unprocessed original scans of blots are shown in Supplementary Fig. 11.

To improve the therapeutic outcome of ABT-263 for SCLC, we sought to identify the best combination strategies that enhance the proapoptotic effect of ABT-737/263. HTS of FDA-approved anti-cancer agents was performed to identify agents that cooperate with ABT-737 to kill ABT-737-resistant SCLC. As reported^10^, DMS53 was sensitive whereas H196 was resistant to ABT-737 (Fig. 1a). Interestingly, H196 was also resistant to the chemotherapeutic agents used to treat SCLC (Fig. 1a). To explore the mechanisms underlying the differential responses of H196 versus DMS53, the levels of BCL-2 family proteins were assessed. The high expression of MCL-1 and low expression of BCL-2 in H196 cells were probably accountable for their resistance to ABT-737 (Fig. 1b). Supporting this notion, knockdown of *MCL-1* sensitized H196 cells to ABT-737 (Supplementary Fig. 1). In addition, H196 expressed lower levels of BID, BIM and PUMA than DMS53 (Fig. 1b), which might contribute to their resistance to ABT-737 and chemotherapeutic agents as reported^5^.

We next performed HTS of 76 FDA-approved anti-cancer agents to identify agents that cooperate with ABT-737 to kill H196 cells (Fig. 1c and Supplementary Data 1). Effector concentration for half-maximum responses (EC50) of each compound from the FDA panel with or without ABT-737 was determined in H196 cells. Nine compounds from the FDA panel were synergistic with ABT-737, resulting in a greater than twofold decrease of EC50 (Fig. 1d). Five of the nine hits were anthracyclines including doxorubicin (Fig. 1d). As doxorubicin has been included in chemotherapeutic regimens for SCLC^21^, we subsequently focused on doxorubicin. The combination of ABT-737 and doxorubicin indeed induced robust apoptosis in H196 cells (Fig. 1e). In contrast, other chemotherapeutic agents used in treating SCLC, including etoposide, cisplatin and camptothecin, failed to induce comparable apoptosis as doxorubicin when combined with ABT-737 (Fig. 1e). These data strongly argue that ABT-737 does not simply lower the apoptotic threshold of standard cytotoxic agents. In fact, only doxorubicin reduced MCL-1 expression (Fig. 1f), which would sensitize H196 cells to ABT-737 based on the *MCL-1* knockdown experiments (Supplementary Fig. 1).

Anthracyclines were reported to be global transcriptional repressors that preferentially affect *MCL-1* due to its short messenger RNA half-life^26^. We confirmed that doxorubicin rapidly decreased *MCL-1* mRNA in H196 cells (Fig. 1g and Supplementary Fig. 2). Of note, doxorubicin did not further reduce *MCL-1* mRNA when combined with actinomycin D, a general transcription inhibitor (Supplementary Fig. 2). Doxorubicin also decreased *BCL-2* and *BCL-X*_*L*_ mRNA but to a lesser extent. Only MCL-1 protein was greatly reduced within 6 h of doxorubicin treatment, likely to be due to the highly labile nature of MCL-1 protein (Fig. 1h). We subsequently assessed a series of SCLC cell lines for their EC50 to ABT-263 (Fig. 1i). H2171 and DMS53 displayed submicromolar EC50 to ABT-263 and were effectively killed by ABT-263 (Fig. 1i). In contrast, H196, SW1271, H82, DMS114 and H446 were relatively resistant to ABT-263. Importantly, the combination of ABT-263 and doxorubicin induced robust apoptosis in these cell lines (Fig. 1j). In summary, our HTS of the FDA-approved anti-cancer agents identified anthracyclines including doxorubicin as a universal combination strategy with ABT-263 in SCLC.

### HTS identifies CDK9 inhibitors that cooperate with ABT-263

**Figure 2:**
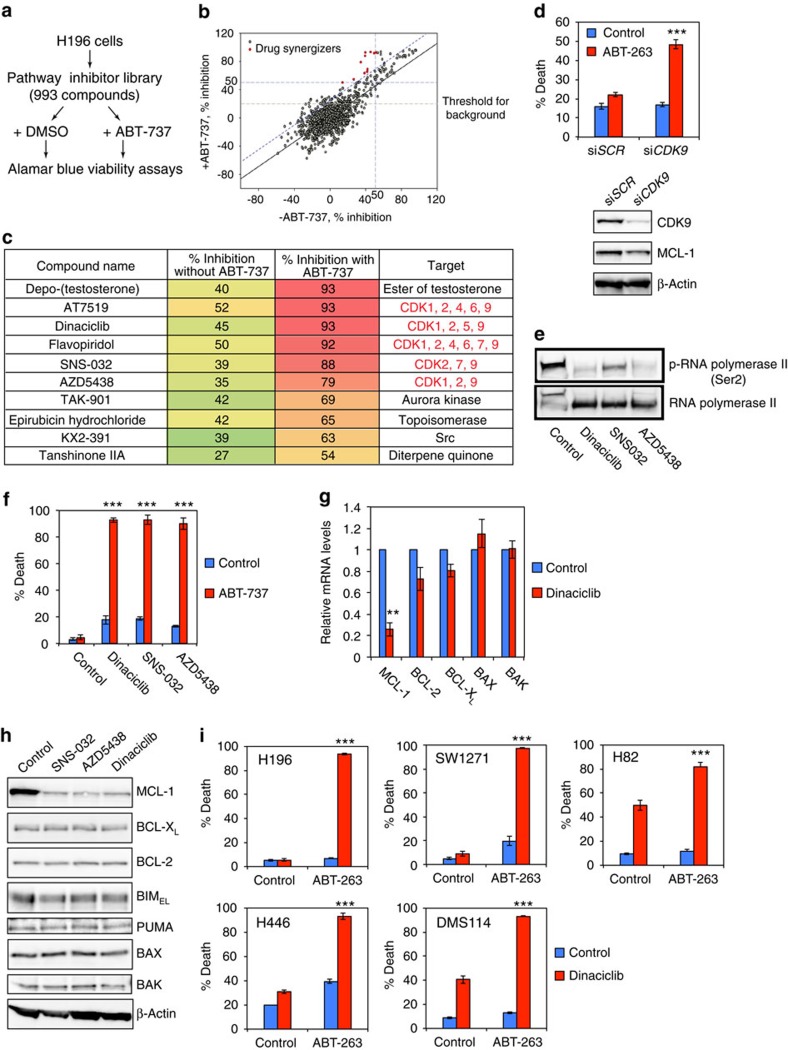
HTS of a small molecule pathway inhibitor library identifies CDK9 inhibitors that enhance the proapoptotic effect of ABT-737. (**a**) A schematic of HTS of the pathway inhibitor library to identify agents that cooperate with ABT-737 to kill H196 cells. H196 cells were used to screen the pathway inhibitor library at 2 μM±1 μM ABT-737. Compounds were screened in duplicate and cell viability was assessed by Alamar Blue assays at 72 h. (**b**) Scatter plot analysis of the average percentage growth inhibition of each compound screened in H196 cells. The compounds that induced ≤50% growth inhibition in the absence of ABT-737 while ≥50% inhibition in the presence of ABT-737 were identified as positive hits (red dots). (**c**) A summary of hit compounds identified by the HTS of the pathway inhibitor library. (**d**) H196 cells, transfected with scrambled siRNA (si*SCR*) or siRNA against *CDK9*, were treated with 1 μM ABT-263 and subjected to cell death or immunoblot analysis. Cell death was quantified by annexin-V staining (mean±s.d., *n*=3). (**e**) H196 cells, treated with the indicated agents for 3 h, were assessed by immunoblot analysis. (**f**) H196 cells were treated with the indicated agents±1 μM ABT-737 for 24 h. Cell death was quantified by annexin-V staining (mean±s.d., *n*=3). (**g**) The mRNA levels of BCL-2 family in H196 cells treated with 10 nM dinaciclib for 3 h were assessed by qRT-PCR. Data were normalized against β-Actin (mean±s.d., *n*=2 independent experiments). (**h**) H196 cells, untreated or treated with the indicated CDK9 inhibitors for 6 h, were assessed by immunoblot analysis. (**i**) The indicated SCLC cell lines were treated with vehicle or dinaciclib (10 nM for H196, H82, and H446, or 20 nM for SW1271 and DMS114) in the absence or presence of 1 μM ABT-263 for 24 h. Cell death was quantified by annexin-V staining (mean±s.d., *n*=3). **P<0.01 and ***P<0.001 (*t*-test). Unprocessed original scans of blots are shown in Supplementary Fig. 11.

Although several targeted therapeutic agents have been demonstrated to enhance the therapeutic effect of ABT-737/263 in preclinical studies, an unbiased approach to identify the best combination strategy for specific cancer types has not been pursued. Accordingly, we extended our HTS to a pathway inhibitor library that is composed of a diverse collection of 993 small molecules against over 200 targets in more than 20 signalling pathways (Supplementary Data 2). H196 cells were treated with each compound from this library with or without ABT-737 (Fig. 2a). Scatterplot analysis of the average percentage growth inhibition identified 11 compounds that conferred more inhibition when combined with ABT-737 (Fig. 2b). Of the 11 hit compounds, 5 were cycline-dependent kinase (CDK) inhibitors that share the common targets of CDK2 and CDK9 (Fig. 2c). PHA-793887 (ref. 27), a CDK inhibitor that mainly targets CDK2, was present in the library but was not identified as a hit. Together, these data suggest that CDK9 is most probably the shared target, which is further supported by the observation that knockdown of CDK9 sensitized H196 cells to ABT-263 (Fig. 2d).

CDK9 is a serine-threonine kinase that forms the catalytic core of p-TEFb complex and, in the presence of cyclin T, phosphorylates Ser2 in the carboxy-terminal domain of RNA polymerase II (Pol II) to stimulate transcription elongation^28^,^29^. CDK9 inhibitors block transcription elongation and preferentially downregulate transcripts with short half-life such as *MCL-1* mRNA^30^,^31^. Indeed, knockdown of CDK9 reduced MCL-1 in H196 (Fig. 2d). We confirmed that dinaciclib, SNS-032 and AZD5438 reduced the Ser2 phosphorylation of Pol II and synergized with ABT-263 to kill H196 cells (Fig. 2e,f). We subsequently focused on dinaciclib because it is more advanced in clinical development^32^. Dinaciclib rapidly reduced *MCL-1* mRNA and protein (Fig. 2g,h). The synergistic effect of ABT-263 and dinaciclib in triggering apoptosis was shown in all tested ABT-263-resistant SCLC cell lines (Fig. 2i). Of note, PHA-793887 neither suppressed the Ser2 phosphorylation of Pol II nor reduced MCL-1 (Supplementary Fig. 3).

Our HTS identified anthracyclines and CDK9 inhibitors as two combination strategies with ABT-263 through downregulation of *MCL-1*. Notably, overexpression of MCL-1 protected H196 cells from apoptosis induced by combined ABT-263 and dinaciclib or doxorubicin (Supplementary Fig. 4), supporting that downregulation of MCL-1 is responsible for the observed synergistic proapoptotic effect. Notably, although >10 different mammalian target of rapamycin (mTOR) inhibitors were present in our library, none were identified as hits. As it was reported that mTOR inhibitors, including rapamycin and AZD8055, sensitize SCLC cell lines to ABT-737/263 (refs 25, 33), we compared the death inducing activity of combined ABT-263 and mTOR inhibitors with that of combined ABT-263 and dinaciclib in four ABT-263-resistant SCLC cell lines (Supplementary Fig. 5). Indeed, combined ABT-263 with dinaciclib was more potent in triggering apoptosis than combined ABT-263 with mTOR inhibitors (Supplementary Fig. 5).

### Prediction of cellular addiction to anti-apoptotic BCL-2s

**Figure 3:**
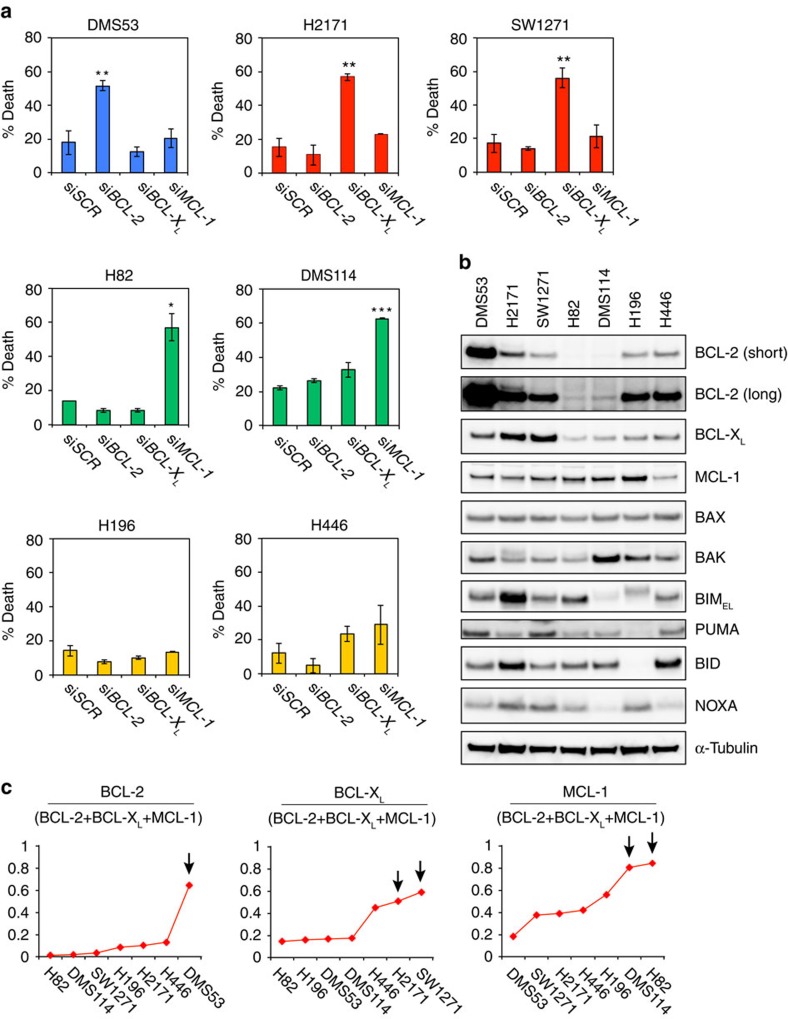
The SCLC cell lines display differential addiction to anti-apoptotic BCL-2 family proteins for survival. (**a**) The indicated SCLC cell lines were transfected with scrambled siRNA (si*SCR*) or siRNA against *BCL-2*, *BCL-X*_*L*_, or *MCL-1*. Cell death was quantified by annexin-V staining (mean±s.d., *n*=3). (**b**) The expression of BCL-2 family proteins in the indicated SCLC cell lines was assessed by immunoblot analysis using the indicated antibodies. (**c**) The protein expression ratios of BCL-2, BCL-X_L_, or MCL-1 to combined BCL-2, BCL-X_L_ and MCL-1 in the indicated SCLC cell lines. The expression of BCL-2, BCL-X_L_, or MCL-1 was normalized against α-Tubulin and the ratio of an individual anti-apoptotic BCL-2 member to all three members was obtained based on two representative immunoblot analyses. Arrows indicate the cell lines that display addiction to BCL-2, BCL-X_L_ or MCL-1 for survival. *P<0.05, **P<0.01 and ***P<0.001 (Student’s *t*-test). Unprocessed original scans of blots are shown in Supplementary Fig. 11.

The findings that doxorubicin and dinaciclib downregulated *MCL-1* and induced apoptosis in H82 and DMS114 as single agents raise the possibility that these cell lines might be dependent on MCL-1 for survival (Figs 1j and 2i). In contrast, DMS53 and H2171 might be dependent on BCL-2 and/or BCL-X_L_ for survival given their sensitivity to ABT-263 (Fig. 1i). DMS114 is unique among the studied SCLC cell lines in that it harbors *MCL1* amplification. We hypothesized that a given SCLC cell line might be addicted to a specific anti-apoptotic BCL-2 member for survival if it predominantly expresses that specific anti-apoptotic BCL-2 member. Conversely, a given SCLC cell line that does not predominantly express a specific anti-apoptotic BCL-2 member might not be addicted to any single anti-apoptotic BCL-2 member. To test this hypothesis, we first determined whether the seven SCLC cell lines display differential addiction to BCL-2, BCL-X_L_ or MCL-1 for survival using RNA interference (RNAi). Knockdown of *MCL-1* but not *BCL-2* or *BCL-X*_*L*_ induced significant apoptosis in H82 and DMS114 (Fig. 3a and Supplementary Fig. 6), supporting that both cell lines are addicted to MCL-1 for survival. In contrast, knockdown of *BCL-2* alone was sufficient to induce significant apoptosis in DMS53 cell line (Fig. 3a and Supplementary Fig. 6), supporting that DMS53 is addicted to BCL-2 for survival. Consistently, ABT-199 effectively killed DMS53 cells (Supplementary Fig. 7). Analogously, H2171 and SW1271 were probably addicted to BCL-X_L_ for survival, because knockdown of *BCL-X*_*L*_ alone was sufficient to induce significant apoptosis (Fig. 3a and Supplementary Fig. 6). Knockdown of *BCL-2*, *BCL-X*_*L*_ or *MCL-1* failed to induce significant apoptosis in H196 and H446, suggesting that these cell lines are not addicted to any single anti-apoptotic BCL-2 member for survival (Fig. 3a and Supplementary Fig. 6). Notably, knockdown of both *BCL-X*_*L*_ and *MCL-1* induced robust apoptosis in H196 (Supplementary Fig. 8), which is consistent with its low expression of BCL-2 (Fig. 1b). To this end, we could divide these seven SCLC cell lines into BCL-2-addicted, BCL-X_L_-addicted, MCL-1-addicted or non-addicted to any single anti-apoptotic BCL-2 member (Fig. 3a).

We next tested whether selective addiction to BCL-2, BCL-X_L_ or MCL-1 for survival is due to the predominant expression of BCL-2, BCL-X_L_ or MCL-1 in a given SCLC cell line by assessing the expression of BCL-2 family proteins (Fig. 3b). The expression of BCL-2, BCL-X_L_ or MCL-1 was normalized against α-Tubulin and the ratio of an individual anti-apoptotic BCL-2 member to all three members was obtained. We found that the ratio of BCL-2 to combined BCL-2, BCL-X_L_ and MCL-1 was highest in BCL-2-addicted DMS53, the ratio of BCL-X_L_ was highest in BCL-X_L_-addicted SW1271 and H2171, and the ratio of MCL-1 was highest in MCL-1-addicted H82 and DMS114 (Fig. 3c). We also compared the mRNA ratios to the protein ratios and found that the mRNA ratios were less predictive than the protein ratios likely due to dysregulated MCL-1 degradation in some cell lines (Supplementary Fig. 9).

### ABT-263 fails to kill BCL-X_L_-addicted cells with low BH3s

**Figure 4:**
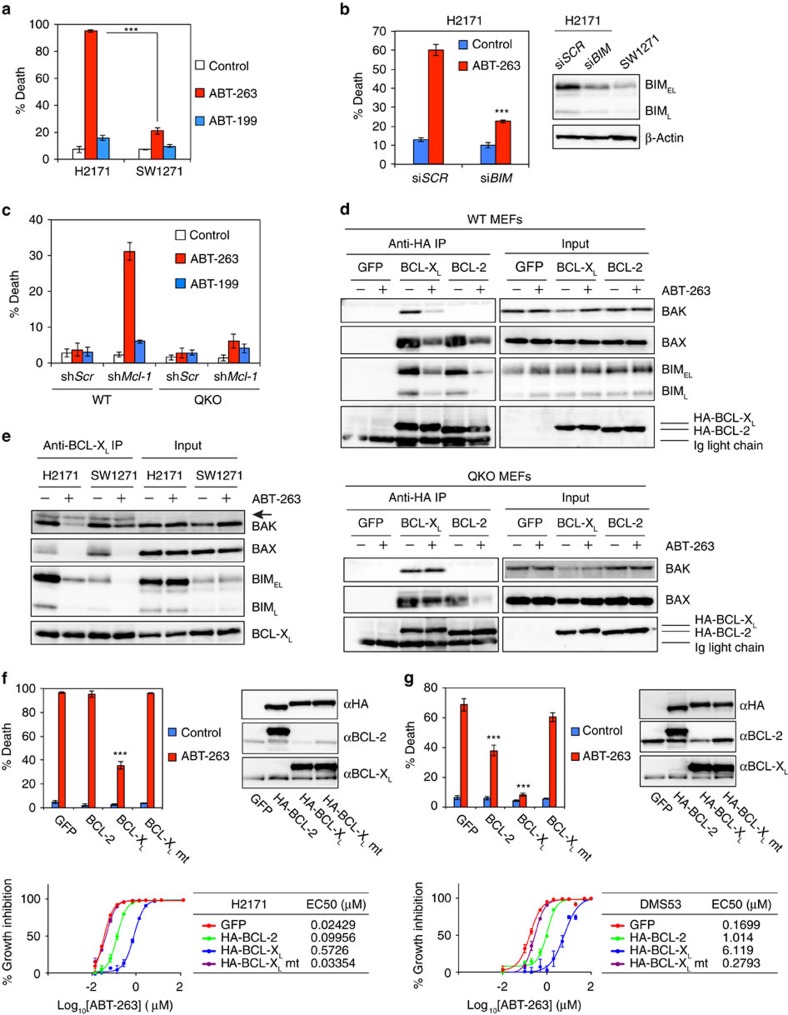
ABT-263 fails to disrupt the interaction between BCL-X_L_ and BAK in the absence of activator BH3s. (**a**) H2171 and SW1271 cells were treated with vehicle, 1 μM ABT-263 or 1 μM ABT-199 for 48 h. Cell death was quantified by annexin-V staining (mean±s.d., *n*=3). (**b**) H2171 cells, transfected with scrambled siRNA (si*SCR*) or siRNA against *BIM*, were untreated or treated with ABT-263 for 24 h. Cell death was quantified by annexin-V staining (mean±s.d., *n*=3). The expression of BIM was assessed by an anti-BIM immunoblot. (**c**) SV40-transformed wild-type or *Bid*^*−/−*^*Bim*^*−/−*^*Puma*^*−/−*^*Noxa*^*−/−*^ QKO MEFs, infected with retrovirus expressing shRNA against luciferase or *Mcl-1*, were untreated or treated with ABT-263 or ABT-199 for 24 h. Cell death was quantified by annexin-V staining (mean±s.d., *n*=3). (**d**) SV40-transformed wild-type or *Bid*^*−/−*^*Bim*^*−/−*^*Puma*^*−/−*^*Noxa*^*−/−*^ QKO MEFs stably expressing GFP, HA-BCL-X_L_ or HA-BCL-2, untreated or treated with ABT-263, were subjected to anti-HA immunoprecipitation. The input (5%) and immunoprecipitates were assessed by immunoblot analysis. (**e**) H2171 and SW1271 cells, untreated or treated with ABT-263, were subjected to anti-HA immunoprecipitation. The input (5%) and immunoprecipitates were assessed by immunoblot analysis. Arrow indicates a cross-reactive band. (**f**) H2171 cells stably expressing GFP, HA-BCL-2, HA-BCL-X_L_, or HA-BCL-X_L_ BH1 mutant were untreated or treated with 1 μM ABT-263 for 24 h. Cell death was quantified by annexin-V staining (mean±s.d., *n*=3). EC50 of ABT-263 in H2171 cells was assessed by CellTiter-Glo assays at 48 h. The expression of HA-tagged BCL-2 and BCL-X_L_ protein was assessed by immunoblot analysis. (**g**) DMS53 cells stably expressing GFP, HA-BCL-2, HA-BCL-X_L_ or HA-BCL-X_L_ BH1 mutant were untreated or treated with 1 μM ABT-263 for 24 h. Cell death was quantified by annexin-V staining (mean±s.d., *n*=3). EC50 of ABT-263 in H2171 cells was assessed by CellTiter-Glo assays at 48 h. The expression of HA-tagged BCL-2 and BCL-X_L_ protein was assessed by immunoblot analysis. ***P<0.001 (Student’s *t*-test). Unprocessed original scans of blots are shown in Supplementary Fig. 11.

We have demonstrated that both SW1271 and H2171 were dependent on BCL-X_L_ for survival based on the RNAi results (Fig. 3a). However, SW1271 displayed the highest EC50 to ABT-263 whereas H2171 showed submicromolar EC50 to ABT-263 (Fig. 1i). To validate these results, viability was determined by annexin-V staining and ABT-199 was included as a control. ABT-263 but not ABT-199 induced robust apoptosis in H2171 (Fig. 4a). In contrast, ABT-263 only induced mild apoptosis in SW1271. The dependency of SW1271 on BCL-X_L_ for survival was validated using a second small interfering RNA (siRNA) against *BCL-X*_*L*_ (Supplementary Fig. 10). Notably, lower expression of BIM and BID was observed in SW1271 cells than in H2171 cells (Fig. 3b). We have previously demonstrated that BID/BIM/PUMA is required for ABT-737/263 to induce apoptosis because ABT-737/263 displaces BID/BIM/PUMA from BCL-2/BCL-X_L_ to activate BAX/BAK indirectly^5^. Moreover, it was reported that BIM expression levels predict the sensitivity of cancer cells to ABT-263 (ref. 25). To determine whether the differential sensitivities of these two cell lines to ABT-263 is caused by different BIM expression, *BIM* was silenced in H2171. Indeed, knockdown of *BIM* conferred resistance to ABT-263 in H2171 (Fig. 4b).

To further confirm that activator BH3s are required for ABT-263 to induce apoptosis, we employed activator BH3-deficient *Bid*^*−/−*^*Bim*^*−/−*^*Puma*^*−/−*^*Noxa*^*−/−*^ quadruple knockout (QKO) mouse embryonic fibroblasts (MEFs)^6^. Knockdown of *Mcl-1* sensitized wild-type MEFs to ABT-263- but not ABT-199-induced apoptosis (Fig. 4c). However, ABT-263 failed to kill QKO cells with *Mcl-1* knockdown (Fig. 4c). This is in stark contrast to our reported knockdown experiments showing that concurrent KD of *Bcl-2*, *Bcl-x*_*L*_ and *Mcl-1* is sufficient to induce autoactivation of BAX/BAK and apoptosis in QKO cells^6^. These data raise a testable hypothesis that ABT-263 may be unable to disrupt the BCL-X_L_/BAK or BCL-X_L_/BAX interaction and thereby fails to kill QKO cells with *Mcl-1* knockdown. To examine this hypothesis, co-immunoprecipitation was performed. In wild-type MEFs, ABT-263 was able to reduce the interaction between BCL-X_L_ and BAX, BAK or BIM (Fig. 4d). In contrast, ABT-263 failed to disrupt the interaction between BCL-X_L_ and BAK in QKO cells, whereas it partially reduced the interaction between BCL-X_L_ and BAX (Fig. 4d). Of note, ABT-263 was able to reduce the interaction between BCL-2 and BAX or BIM in both wild-type and QKO cells (Fig. 4d). As reported^6^,^34^,^35^, BCL-2 only interacted with BAX but not BAK. Together, these data suggest that ABT-263 releases BIM and other activator BH3s from BCL-2 and BCL-X_L_ to disrupt the BCL-X_L_/BAK interaction indirectly. Accordingly, we examined whether ABT-263 has differential effects on disrupting the BCL-X_L_/BAK interaction in H2171 versus SW1271 cells. ABT-263 had minimal effect on the interaction between BCL-X_L_ and BAK in SW1271 with low BIM expression, whereas ABT-263 greatly reduced this interaction in H2171 with high BIM expression (Fig. 4e). This is consistent with the notion that BIM is required to disrupt the BCL-X_L_/BAK interaction after its displacement from BCL-X_L_ or BCL-2 by ABT-263. Collectively, ABT-263 is likely a poor BCL-X_L_ inhibitor in cells with low activator BH3s because it is unable to directly disrupt the BCL-X_L_/BAK interaction.

### Overexpression of BCL-X_L_ confers resistance to ABT-263

We have reported that BCL-2, BCL-X_L_ and MCL-1 inhibit bimodal activation of BAX/BAK by sequestering activator BH3s and ‘BH3-exposed’ BAX/BAK monomers, respectively^6^. BCL-X_L_ is superior to BCL-2 in preventing apoptosis, because BCL-X_L_ but not BCL-2 can bind and inhibit BAK^6^. Given that ABT-263 could not directly disrupt the BCL-X_L_/BAK interaction, we hypothesized that overexpression of BCL-X_L_ might provide more resistance to ABT-263 than overexpression of BCL-2 based on the unique capacity of BCL-X_L_ to directly bind and inhibit ‘BH3-exposed’ BAK monomers. Indeed, overexpression of HA-tagged BCL-X_L_ but not BCL-2 inhibited ABT-263-induced apoptosis in BCL-X_L_-addicted H2171 (Fig. 4f). Notably, the BCL-X_L_ BH1 mutant (G138E/R139L/I140N) that is unable to sequester proapoptotic BCL-2 members failed to protect H2171 (Fig. 4f). BCL-X_L_ overexpression resulted in a ∼24-fold increase in the EC50 of ABT-263 whereas BCL-2 overexpression resulted in a ∼4-fold increase in H2171 (Fig. 4f). Furthermore, overexpression of BCL-X_L_ in BCL-2-addicted DMS53 completely inhibited ABT-263-induced apoptosis, whereas overexpression of BCL-2 only partially inhibited apoptosis (Fig. 4g). BCL-X_L_ overexpression resulted in a ∼36-fold increase in the EC50 of ABT-263, whereas BCL-2 overexpression resulted in a ∼6-fold increase in DMS53 (Fig. 4g). In both H2171 and DMS53, BCL-X_L_ overexpression increased the EC50 of ABT-263 by sixfold in comparison with BCL-2 overexpression. Overall, these results suggest that overexpression of BCL-X_L_ might be a potential resistance mechanism to ABT-263 due to the inability of ABT-263 to disrupt the BCL-X_L_/BAK interaction.

### JQ1 neither downregulates *MCL-1* nor cooperates with ABT-263

**Figure 5:**
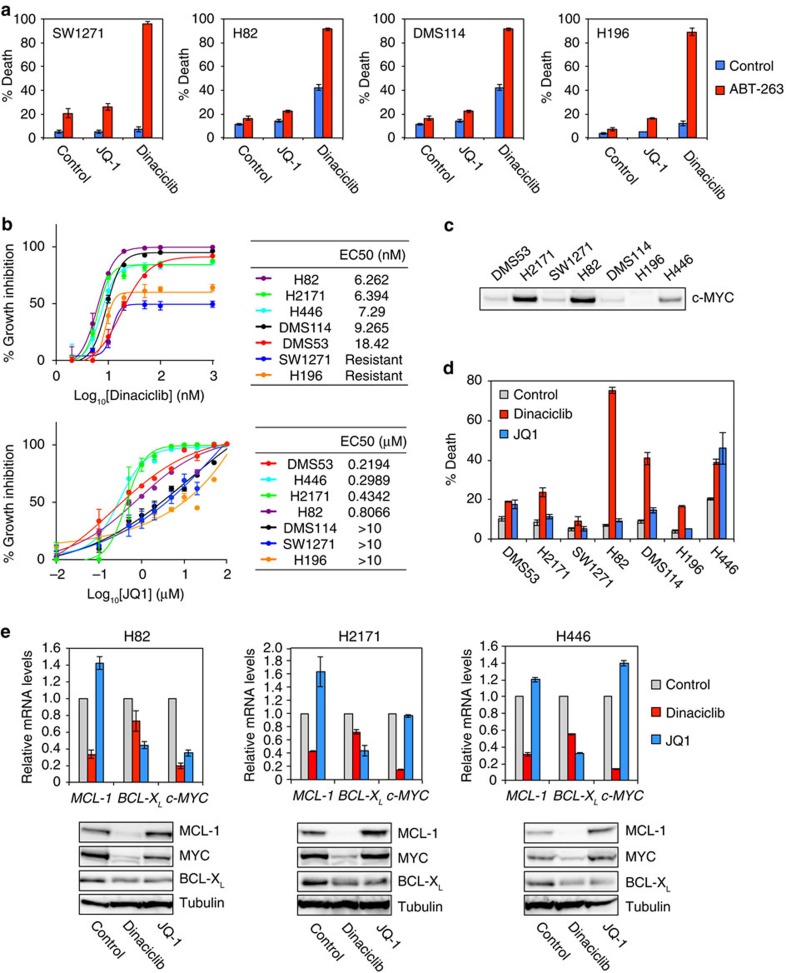
Dinaciclib but not JQ1 synergizes with ABT-263 to kill SCLC cells. (**a**) The indicated SCLC cell lines were treated with vehicle, 1 μM JQ1, or dinaciclib (10 nM for H196 and H82, or 20 nM for SW1271 and DMS114) in the absence or presence of 1 μM ABT-263 for 24 h. Cell death was quantified by annexin-V staining (mean±s.d., *n*=3). (**b**) A summary of EC50s of dinaciclib and JQ1 in SCLC cell lines. The indicated SCLC cell lines were untreated or treated with increasing concentrations of dinaciclib or JQ1. Cell viability was assessed by CellTiter-Glo assays at 72 h. (**c**) The expression of c-MYC in the indicated SCLC cell lines was assessed by an anti-c-Myc immunoblot. (**d**) The indicated SCLC cell lines were treated with vehicle, 1 μM JQ1, or 20 nM dinaciclib for 24 h. Cell death was quantified by annexin-V staining (mean±s.d., *n*=3). (**e**) H82, H2171, and H446 cells, treated with vehicle, 20 nM dinaciclib, or 1 μM JQ1 for 3 h, were assessed by qRT-PCR. Data were normalized against β-actin (mean±s.d., *n*=2 independent experiments). H82, H2171, and H446 cells, treated with vehicle, dinaciclib, or JQ1 for 24 h, were assessed by immunoblot analysis using the indicated antibodies. Unprocessed original scans of blots are shown in Supplementary Fig. 11.

BRD4, a member of the BET family, is involved in the control of transcriptional elongation by Pol II through its recruitment of p-TEFb. Hence, we examined whether the BET inhibitor JQ1 (ref. 36) could recapitulate the effect of CDK9 inhibitors. JQ1 had no apparent single agent activity and failed to synergize with ABT-263 (Fig. 5a). Furthermore, there was no clear correlation of EC50s between dinaciclib and JQ1 (Fig. 5b), for example, DMS114 was sensitive to dinaciclib but not JQ1. Interestingly, three SCLC cell lines with *c-MYC* amplification, including H82, H2171 and H446, had the lowest EC50 to dinaciclib, which is consistent with a recent identification of CDK9 inhibition as a therapeutic strategy for c-MYC-overexpressing hepatocellular carcinoma^37^. c-MYC expression was indeed higher in *c-MYC*-amplified SCLC cell lines (Fig. 5c). As the EC50 was determined by growth inhibition, it might reflect anti-proliferative rather than death-inducing activity. Accordingly, cell death was quantified by annexin-V staining (Fig. 5d). Potent induction of apoptosis by dinaciclib at both 10 nM (Fig. 2i) and 20 nM (Fig. 5d) was observed in H82 and DMS114 cells that are addicted to MCL-1. In contrast, JQ1 failed to induce apoptosis in both H82 and DMS114 (Fig. 5d). JQ1 only induced marked apoptosis in H446 cells that are not addicted to any single anti-apoptotic member (Fig. 5d). Significant apoptosis was also observed in H446 cells treated with 20 nM but not 10 nM dinaciclib (Figs 2i and 5d). Dinaciclib appeared to inhibit proliferation rather than inducing apoptosis in another *c-MYC*-amplified H2171 cells that are addicted to BCL-X_L_ (Fig. 5b,d).

To this end, we envisioned that the reason why JQ1 fails to synergize with ABT-263 is most likely due to its inability to downregulate *MCL-1*. JQ1 was shown to prevent the binding of BRD4 to super-enhancers and cause transcription elongation defects that preferentially impact genes with super-enhancers, such as *c-MYC* and *BCL-X*_*L*_^38^. We next examined the effect of dinaciclib and JQ1 on the expression of c-MYC, MCL-1 and BCL-X_L_ in H82, H2171 and H446 cells (Fig. 5e). Dinaciclib greatly reduced the expression of both MCL-1 and c-MYC in these cell lines (Fig. 5e). In contrast, JQ1 induced MCL-1 (Fig. 5e), consistent with its inability to either kill MCL-1-addicted cells or synergize with ABT-263. Intriguingly, c-MYC was reduced, unaffected or induced by JQ1 in these cell lines (Fig. 5e), which is similar to what has been reported for lung adenocarcinoma^39^. The anti-proliferative effect of JQ1 for these cell lines might be unrelated to c-MYC, as suggested for lung adenocarcinoma^39^. Collectively, our data suggest that dinaciclib will probably have single-agent anti-tumour activity against a subset of SCLC that either carry *c-MYC* amplification or are MCL-1-addicted.

### Effective combination strategies for SCLC using ABT-199

**Figure 6:**
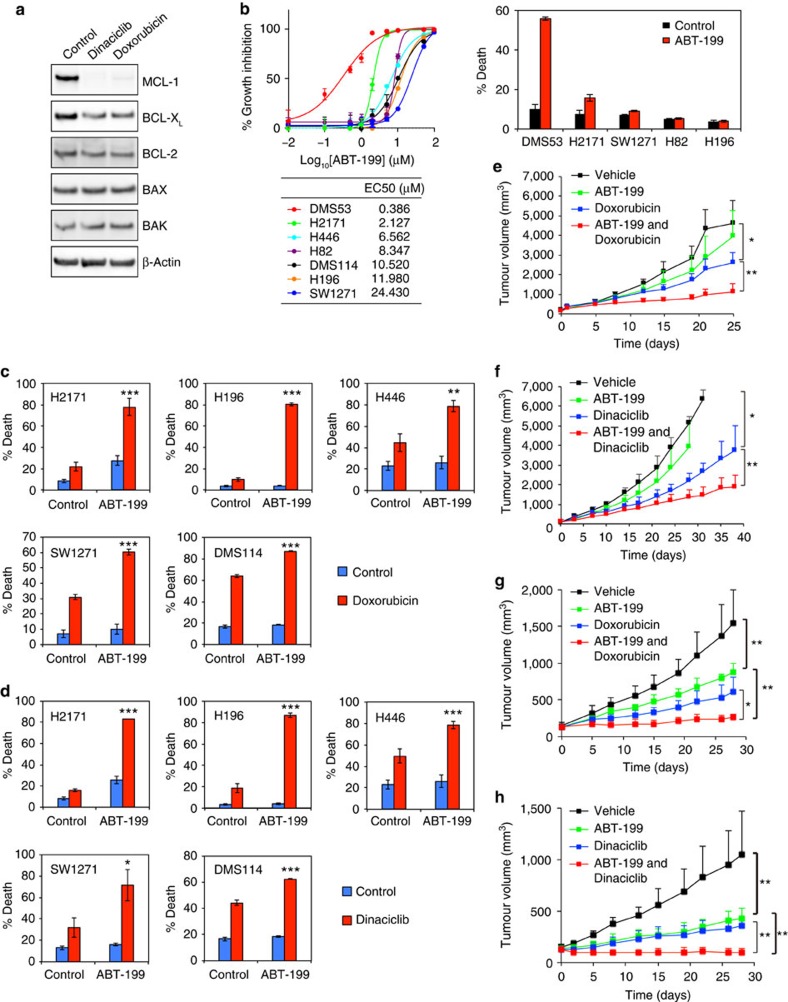
Doxorubicin or dinaciclib cooperates with ABT-199 to kill SCLC cells. (**a**) H196 cells, treated with dinaciclib or doxorubicin for 18 h, were assessed by immunoblot analysis. (**b**) A summary of EC50s of ABT-199. Cell death response to 1 μM ABT-199 was quantified by annexin-V staining at 48 h (mean±s.d., *n*=3). (**c**) The indicated cell lines, treated with 2 μM doxorubicin±1 or 2 μM ABT-199, were quantified by annexin-V staining at 24 h for H2171 and H446 or at 48 h for H196 and SW1271 (mean±s.d., *n*=3). (**d**) The indicated cell lines, treated with 10–20 nM dinaciclib±1 or 2 μM ABT-199, were quantified by annexin-V staining at 24 h for H2171 and H446, at 48 h for H196, or at 72 h for SW1271 (mean±s.d., *n*=3). *P<0.05, **P<0.01 and ***P<0.001 (Student’s *t*-test). (**e**) NSG mice bearing H446 xenografts were treated with vehicle (*n*=6), ABT-199 (100 mg kg^−1^, *n*=8), doxorubicin (2 mg kg^−1^, *n*=8), or combined ABT-199 and doxorubicin (*n*=8). tumour volumes were measured twice weekly by caliper (mean±s.d.). *P=0.0022 and **P<0.0001 (two-way analysis of variance (ANOVA)). (**f**) NSG mice bearing H446 xenografts were treated with vehicle (*n*=8), ABT-199 (100 mg kg^−1^, *n*=8), dinaciclib (20 mg kg^−1^, *n*=8), or combined ABT-199 and dinaciclib (*n*=8). *P=0.0002; **P=0.0034 (two-way ANOVA). (**g**) NSG mice bearing patient-derived SCLC xenografts were treated with vehicle (*n*=5), ABT-199 (100 mg kg^−1^, *n*=4), doxorubicin (2 mg kg^−1^, *n*=6), or combined ABT-199 and doxorubicin (*n*=4). *P=0.0029; **P<0.0001 (two-way ANOVA). (**h**) NSG mice bearing patient-derived SCLC xenografts were treated with vehicle (*n*=6), ABT-199 (100 mg kg^−1^, *n*=6), dinaciclib (30 mg kg^−1^, *n*=6), or combined ABT-199 and dinaciclib (*n*=8). **P<0.0001 (two-way ANOVA). Unprocessed original scans of blots are shown in Supplementary Fig. 11.

Although doxorubicin and dinaciclib had more prompt and profound effects on reducing the expression of MCL-1 than BCL-2 and BCL-X_L_, we found that prolonged treatment of SCLC cells with these agents also partially reduced BCL-X_L_ (Fig. 6a). These findings suggested a potentially effective therapeutic strategy in which combined ABT-199 with doxorubicin or dinaciclib might be sufficient to trigger apoptosis in SCLC. To test this idea, we first assessed EC50 of ABT-199 in various SCLC cell lines (Fig. 6b). Consistent with our characterization of DMS53 cells as BCL-2-addicted, DMS53 had the lowest EC50 to ABT-199 and was the only cell line that underwent potent apoptosis following ABT-199 treatment (Fig. 6b). Importantly, combined ABT-199 with doxorubicin or dinaciclib induced robust apoptosis in all tested SCLC cells including those ABT-263-resistant ones (Fig. 6c,d).

We next examined the *in vivo* anti-tumour efficacy of combined ABT-199 and doxorubicin or dinaciclib in the H446 xenograft model. Of note, H446 harbours mutations of *RB1* and *TP53* as well as *c-MYC* amplification, all of which are pathognomonic mutations of SCLC. Consistent with our *in vitro* study (Fig. 6c), monotherapy with ABT-199 or doxorubicin had minimal and modest effects on tumour growth, respectively (Fig. 6e). Importantly, combined ABT-199 with doxorubicin markedly inhibited tumour growth without overt signs of toxicity (Fig. 6e). Similarly, combined ABT-199 with dinaciclib was superior to the respective monotherapy and greatly suppressed tumour growth (Fig. 6f). The therapeutic effects of combined ABT-199 with doxorubicin or dinaciclib were further demonstrated in a patient-derived SCLC xenograft (Fig. 6g,h). Collectively, these data suggest that combined ABT-199 with doxorubicin or dinaciclib could provide effective therapeutic strategies for SCLC.

## Discussion

**Figure 7:**
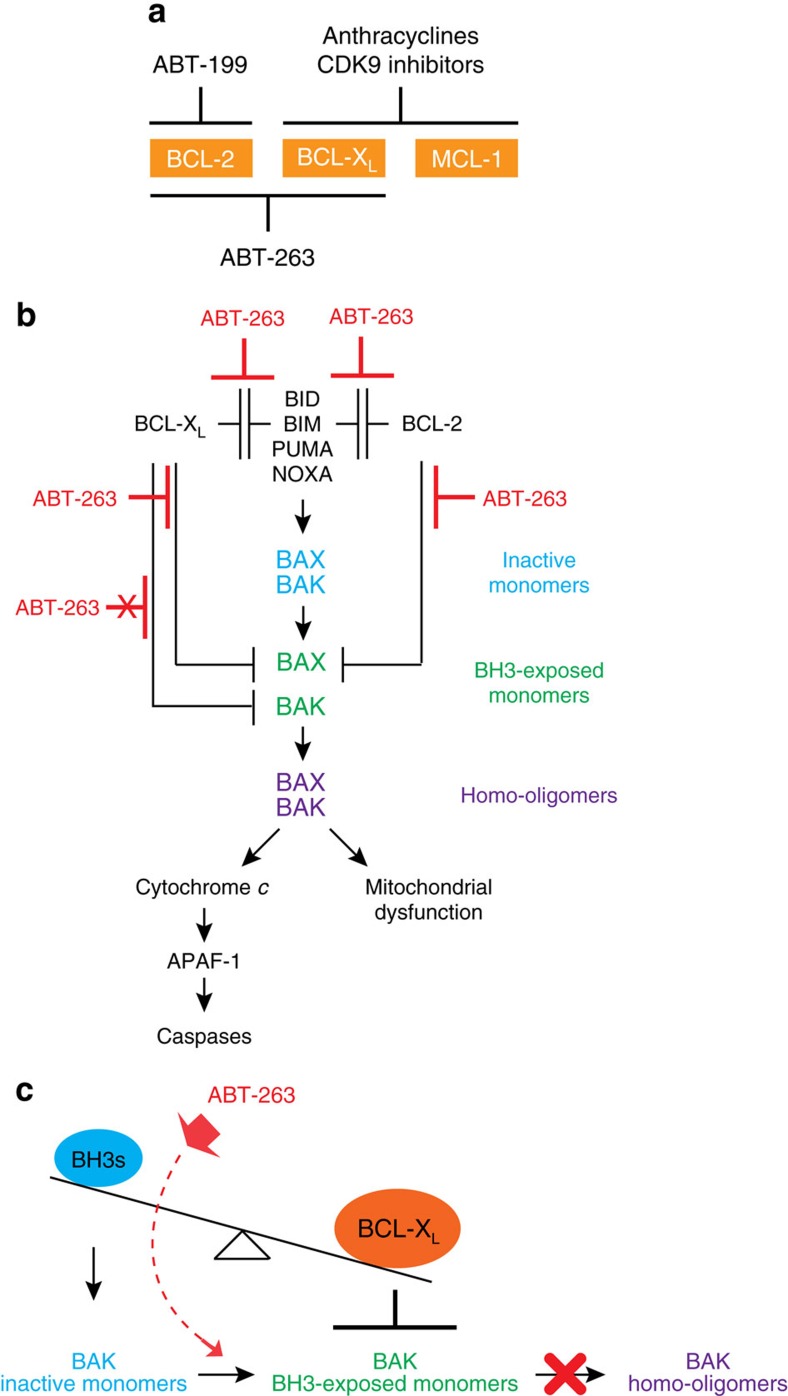
Schematic depiction of mechanism-guided targeting of the BCL-2 family for cancer therapy. (**a**) Differential targeted inhibition of BCL-2, BCL-X_L_ or MCL-1 by ABT-199, ABT-263, anthracyclines, or CDK9 inhibitors. (**b**) A schematic depicts the interconnected hierarchical model of cell death regulation by the BCL-2 family proteins and differential inhibitory activity of ABT-263 against BCL-2 versus BCL-X_L_. ABT-263 prevents BCL-2 and BCL-X_L_ from sequestering both activator BH3s and BAX. In contrast, ABT-263 fails to prevent BCL-X_L_ from sequestering BAK. (**c**) BCL-X_L_ overabundance confers resistance to ABT-263 where BCL-X_L_ prevents the ‘BH3-exposed’ BAK monomers from undergoing homo-oligomerization.

Evading cell death is one of the hallmarks of cancer^40^. Oncogenic transformation that drives uncontrolled cell-cycle progression often activates innate tumour-suppressive checkpoints that trigger apoptosis^41^. One plausible explanation is that BIM and PUMA are transcriptionally activated by E2F1 (ref. 42), a key cell cycle driver upon malignant transformation. To abrogate apoptotic checkpoints, cancer cells often overexpress anti-apoptotic BCL-2 family proteins that sequester upregulated BIM and PUMA. Hence, many cancer cells are likely ‘primed’ to undergo apoptosis upon the administration of BAD and NOXA mimetics that displace BIM/PUMA from BCL-2/BCL-X_L_ and MCL-1, respectively, to activate BAX/BAK^12^,^13^,^14^. As most normal cells do not have excessive BIM/PUMA readily complexed with anti-apoptotic BCL-2 members, they are less ‘primed’ and less susceptible to BAD or NOXA mimetics. In fact, clinical efficacy of ABT-263 (navitoclax) and ABT-199 (venetoclax) has been demonstrated in clinical trials^12^,^13^,^14^,^18^. In the era of precision medicine, it is paramount to identify and select patients who will respond to ABT-263 or ABT-199. Given that clinically applicable MCL-1 inhibitors are not yet available, strategies such as combination therapy to improve the therapeutic efficacy of ABT-263/199 are needed. Using protein expression ratios, we were able to predict the dependency of SCLC cell lines on BCL-2, BCL-X_L_ or MCL-1 for survival. Moreover, we have identified two combination strategies through unbiased HTS, that is, combining ABT-263 with anthracyclines such as doxorubicin or with CDK9 inhibitors such as dinaciclib, which induced potent apoptosis in all examined SCLC cell lines regardless of their dependency on different anti-apoptotic BCL-2 members for survival (Fig. 7a). We further demonstrated that ABT-199 could replace ABT-263 for these two combination strategies, because both doxorubicin and dinaciclib also partially reduced BCL-X_L_ (Fig. 7a). The combination strategies using ABT-199 are expected to reduce the toxicity associated with ABT-263.

Mechanistically, therapeutic modalities that either reduce MCL-1 mRNA/protein or induce proapoptotic BCL-2 family proteins could synergize with ABT-263 to induce apoptosis. Examples include anthracyclines and CDK9 inhibitors that downregulate *MCL-1* (refs 26, 30, 31), histone deacetylase and MEK inhibitors that induce BIM^43^,^44^,^45^, and phosphatidyl inositol 3-kinase inhibitors that induce PUMA^44^. Cancer type-specific synergizers of ABT-263 will probably prevail due to differing aberrant signalling pathways that coalesce into unique vulnerabilities. MEK and phosphatidyl inositol 3-kinase inhibitors were not identified in our HTS of SCLC in which kinase mutations are less common. Instead, anthracyclines and CDK9 inhibitors with transcriptional repressor function were identified as top hits in SCLC where near universal inactivation of p53 and RB1 directly impacts the transcriptional landscape.

It was reported that oncogenic and lineage-specific transcription factors are associated with super-enhancers in SCLC^46^, and that JQ1 preferentially inhibits the transcription of genes with super-enhancers^38^, suggesting that JQ1 may provide a therapeutic effect for SCLC. However, JQ1 neither downregulated *MCL-1* nor sensitized SCLC cell lines to ABT-263 and the EC50s of JQ1 did not correlate with those of dinaciclib in the examined SCLC cell lines. Interestingly, we found that SCLC cell lines with *c-MYC* amplification were more sensitive to dinaciclib and dinaciclib alone was sufficient to induce apoptosis in MCL-1-addicted SCLC cell lines. Together, our data suggest that CDK9 inhibitors may have single-agent anti-tumour activity against a subset of SCLC that either carry *c-MYC* amplification or are MCL-1-addicted. It was reported that SCLC is highly sensitive to a covalent CDK7 inhibitor, THZ1 (ref. 46). During transcription initiation, the carboxy-terminal domain of Pol II is phosphorylated on Ser5 by the TFIIH-associated kinase CDK7, and as Pol II elongates, Ser2 is increasingly phosphorylated by CDK9/p-TEFb, while Ser5 phosphorylation is gradually removed by phosphatases^29^. In fact, THZ1 was shown to reduce the phosphorylation of both Ser5 and Ser2 in SCLC^46^. It is possible that downregulation of MCL-1 is responsible for the death-inducing activity of THZ1 in SCLC.

Using RNAi to determine the dependency on individual anti-apoptotic BCL-2 members for survival, we have classified SCLC cell lines into BCL-2-addicted, BCL-X_L_-addicted, MCL-1-addicted or non-addicted to any single anti-apoptotic BCL-2 member. As expected, the BCL-2-addicted SCLC cells were sensitive to both ABT-199 and ABT-263, whereas the MCL-1-addicted SCLC cells were insensitive to both. Surprisingly, the two BCL-X_L_-addicted SCLC cell lines showed differential sensitivity to ABT-263 due to different BIM expression. We have previously established an interconnected hierarchical model that explains how the intricate interplays between three BCL-2 subfamilies dictate cellular survival versus death^6^ (Fig. 7b). Anti-apoptotic BCL-2, BCL-X_L_ and MCL-1 inhibit apoptosis through sequestering activator BH3s from activating BAX/BAK, providing a frontline protection against apoptotic insults^2^,^3^,^4^. In addition, BCL-2, BCL-X_L_ and MCL-1 can sequester partially activated, BH3-exposed, BAX/BAK monomers to prevent the homo-oligomerization of BAX/BAK (ref. 6), serving as a fail-safe mechanism or the second line of defense. We showed that ABT-263 prevented BCL-2 and BCL-X_L_ from sequestering BIM and BAX, but failed to directly disrupt the interaction of BCL-X_L_ and BAK (Figs 4 and 7b). The inability of ABT-263 to breach the second-line defense of BCL-X_L_ against BAK foretells major limitations to its use. ABT-263 is probably ineffective in treating BCL-X_L_-addicted cancers with low expression of activator BH3s such as BIM, as well as cancers with BCL-X_L_ overabundance, which prevents the BH3-exposed BAK monomers from undergoing homo-oligomerization (Fig. 7c). It is intriguing that ABT-263 fails to directly dissociate the BCL-X_L_/BAK complexes given the high binding affinity of ABT-263 to the hydrophobic dimerization groove of BCL-X_L_. BAX appears to differ from BAK because ABT-263 could disrupt the interaction between BAX and BCL-X_L_ or BCL-2 (Figs 4 and 7b). It is possible that additional contact sites between BCL-X_L_ and BAK further strengthen the BH3-in-groove heterodimers and/or prevent the accessibility of ABT-263 to the dimerization pocket. Alternatively, the lipid milieu of MOM where BCL-X_L_ and BAK reside may modulate their interaction. Nonetheless, targeting the interaction between BCL-X_L_ and BAK is essential for the treatment of ‘poorly primed’ yet BCL-X_L_-addicted cancer (Fig. 7c), which remains unaddressed by ABT-263.

A potential limitation for our prediction model extends to cancers with high expression of BCL2A1 or BCL-W. Notably, SCLC has the lowest expression of BCL2A1 among the 1,000 cell lines analysed by the Cancer Cell Line Encyclopedia project^47^. The predominant association of a specific anti-apoptotic BCL-2 member with BIM and BAK has been shown to predict dependency for survival in multiple myeloma and AML, respectively^48^,^49^. Thus far, most of the prediction methods have been focused on the identification of ABT-263-responsive cancers. BH3 profiling is one such example, which evaluates apoptotic sensitivity or ‘mitochondrial priming’ of cancer cells by measuring whether BH3 domain peptides induce MOMP^50^. Of note, our prediction method may not be applicable to highly primed cancer such as hematological malignancies that highly express BIM as revealed by the Cancer Cell Line Encyclopedia project. Highly primed cancers may be sensitive to the knockdown of any individual anti-apoptotic BCL-2 member even though they predominantly express one anti-apoptotic BCL-2 member. Accordingly, highly primed cancers may be sensitive to ABT-263 even though they are MCL-1-dependent, which has been shown in multiple myeloma^48^.

Our study establishes a framework for identifying and targeting the differential addiction to anti-apoptotic BCL-2 family proteins in SCLC, which could potentially be applied to other cancer types. Combined inhibition of BCL-2, BCL-X_L_ and MCL-1 through mechanism-guided combination therapy will provide the most effective and a potentially universal therapeutic strategy to eradicate cancer cells through apoptotic induction. We presented two such strategies, that is, the combinations of ABT-199 and doxorubicin or dinaciclib. Furthermore, we have identified the inability of ABT-263 to prevent BCL-X_L_ from sequestering BAK as a major limitation of its application in cancer therapy. A better understanding of the BCL-X_L_/BAK interaction may aid in the development of a better BCL-X_L_ inhibitor, which is critical for the advancement of targeted therapies against BCL-2 family, as is the development of a clinically applicable MCL-1 inhibitor. Overall, this study demonstrates the feasibility of exploiting the interconnected hierarchical cell death model for the development of specific therapeutic strategies aimed at direct activation of mitochondrial apoptosis in susceptible cancer cells.

## Methods

### Cell culture and viability assay

NCI-H196, SW1271, NCI-H2171, NCI-H446, DMS53 and DMS114 were obtained from the American Type Culture Collection. H82 was obtained from Dr Charles Rudin at Memorial Sloan Kettering Cancer Center. All cell lines were cultured according to the recommendations of the American Type Culture Collection. SV40-transformed wild-type or *Bid*^−/−^ *Bim*^−/−^*Puma*^−/−^*Noxa*^−/−^ MEFs were described previously^6^. Cell death was quantified by Annexin V (BioVison) staining, followed by flow cytometric analyses using an LSRFortessa (BD Biosciences). Data were analysed using FACSDiva (BD Biosciences). For EC_50_ determination, cell viability was assessed by the CellTiter-Glo luminescence assays (Promega) using 96-well plates and a luminescent plate reader (SpectraMax M2e, Molecular Devices). EC_50_ value was calculated using Prism software (GraphPad). *P*-values for statistical analyses were obtained with Student’s *t*-test. The chemicals used are as follows: ABT-737 (Selleck Chemicals); ABT-263 (AbbVie and Selleck Chemicals); ABT-199 (AbbVie and Selleck Chemicals); dinaciclib (Selleck Chemicals); SNS-032 (Selleck Chemicals); AZD5438 (Selleck Chemicals); JQ1 (Selleck Chemicals); doxorubicin (Sigma); etoposide (Sigma); cisplatin (Sigma); camptothecin (Sigma); rapamycin (Sigma); everolimus (Selleck Chemicals); AZD8580 (Selleck Chemicals); FDA-panel for HTS (Sigma); actinomycin D (Sigma); PHA-793887 (Selleck Chemicals); and pathway inhibitors for HTS (Selleck Chemicals).

### High-throughput screening

HTS was performed by the HTS Core Facility at Memorial Sloan Kettering Cancer Center using an automated 384-well platform. H196 cells were screened against FDA-approved anti-cancer agents (Sigma) and a pathway inhibitor library (Selleck Chemicals) in combination with ABT-737. For the screening of FDA panel, H196 cells were treated with each compound at 12 twofold serial dilution doses starting from 10 μM in the absence or presence of 1 μM ABT-737. Compounds were screened in duplicate and the growth inhibition was assessed by Alamar Blue assays at 3 days post drug treatment. The average Z’ factor for the assay robustness was 0.45. Dose response curves for the FDA panel were fitted using logistic four-parameter sigmoid regression equations and EC50 values were calculated by Sigma-plot (Systat Software). For the screening of pathway inhibitors, H196 cells were treated with each compound at 2 μM in the absence or presence of 1 μM ABT-737. Compounds were screened in duplicate and the growth inhibition was assessed by Alamar Blue assays at 3 days post drug treatment. The average Z’ factor for the assay robustness was 0.48. Scatterplot analysis of the average percentage growth inhibition was performed to identify compounds that synergized with ABT-737 to kill H196 cells.

### RNA interference

siRNA oligos were purchased from Ambion Silencer Select oligos (Applied Biosystems), the sequences of which are summarized in Supplementary Data 3. siRNA oligos were reverse transfected with Lipofectamine RNAiMAX (Invitrogen) to a final concentration of 10 nM.

### Reverse transcription and quantitative PCR

Total RNA was extracted from cells with TRIZOL (Life Technologies) according to the manufacturer’s instruction. Reverse transcription was performed with oligo-dT plus random decamer primers (Ambion) with SuperScript II (Life Technologies). The primers are summarized in Supplementary Data 3. Quantitative PCR was performed with SYBR Green Master Mix (Applied Biosystems) in duplicate with the indicated gene-specific primers. Quantitative PCR was performed on an ABI Prism 7,300 sequence detection system (Applied Biosystems). Data were analysed by normalization against β-actin.

### Immunoblot analysis and immunoprecipitation

Cells were lysed in RIPA buffer. Protein concentration was determined by BCA kit (Pierce). Twenty-five to 50 μg of proteins were resolved by 10% NuPAGE (Life Technologies), transferred onto polyvinylidene difluoride membrane (Immobilon-P, Millipore). Antibody detection was accomplished using enhanced chemiluminescence method (Western Lightning, PerkinElmer) and LAS-3000 Imaging system (FUJIFILM). Antibodies used for immunoblot analysis are listed as followed: anti-BAK (NT, Millipore), anti-BAX (N-20, Santa Cruz Biotechnology), anti-BIM (C34C5, Cell Signaling Technology), anti-PUMA (no. 4976, Cell Signaling Technology), anti-NOXA (ab13654, Abcam), anti-BID (FL-195, Santa Cruz Biotechnology), anti-BCL-2 (6C8), anti-BCL-X_L_ (7B2.5), anti-MCL-1 (S-19, Santa Cruz Biotechnology), anti-phospho RNA Pol II S2 (A300-654A, Betyl Laboratories), anti-CDK9 (C-20, Santa Cruz Biotechnology), anti-MYC (06–340, Millipore), anti-α-Tubulin (T6199, Sigma) and anti-β-actin (A-1978, Chemicon). To quantify protein expression ratios, immunoblots were assessed by ImageJ (v. 1.46r). The relative expression ratios of BCL-2, BCL-X_L_, or MCL-1 to combined BCL-2, BCL-X_L_ and MCL-1 were calculated using the quantified intensity of each protein normalized against α-Tubulin. Co-immunoprecipitation was performed as described^6^ using the anti-HA (12CA5) or anti-BCL-X_L_ (7B2.5) antibodies and analysed by 10% NuPAGE (Life Technologies) and immunoblots.

### Xenograft studies

Animal experiments were performed in accordance with the MSKCC Institutional Animal Care and Use Committee. Sex-matched 7–8 week-old *NOD*/*SCID*/IL2Rγ^null^ (NSG) mice (Jackson Laboratories) were injected subcutaneously with 3 × 10^6^ H446 cells in 0.2 ml 50% Matrigel (BD Biosciences). The PDX model (ECLC9) was derived from a patient previously treated with carboplatin and etoposide in accordance with the MSKCC Institutional Review Board approved tissue collection protocol with informed consent from the patient and propagated in NSG mice. Tumour growth was monitored twice weekly by calipers (volume=length × width^2^/2). When tumours reached ∼150 mm^3^, tumour-bearing mice were randomized according to tumour volume. ABT-199 (100 mg kg^−1^) was administered by oral gavage at a weekly schedule of 5 days on and 2 days off. ABT-199 was formulated in 10% ethanol, 30% polyethylene glycol 400 and 60% Phosal 50 PG. Doxorubicin was formulated in PBS and administered intravenously once weekly at 2 mg kg^−1^. Dinaciclib was formulated in 20% hydroxypropyl β-cyclodextrin and administered intraperitoneally twice weekly at 20 or 30 mg kg^−1^. *P*-values for statistical analyses were obtained with two-way analysis of variance.

### Data availability

All relevant data not presented in the main figures or Supplementary Information is available from the corresponding author upon request.

## Additional information

**How to cite this article:** Inoue-Yamauchi, A. *et al*. Targeting the differential addiction to anti-apoptotic BCL-2 family for cancer therapy. *Nat. Commun.* **8,** 16078 doi: 10.1038/ncomms16078 (2017).

**Publisher’s note:** Springer Nature remains neutral with regard to jurisdictional claims in published maps and institutional affiliations.

## Supplementary Material

Supplementary Information

Supplementary Data 1

Supplementary Data 2

Supplementary Data 3
