# Supplementary material for: Targeting the differential addiction to anti-apoptotic BCL-2 family for cancer therapy
Source: Nat Commun. 2017 Jul 17;8:16078. doi: 10.1038/ncomms16078 (PMC5520052; doi:10.1038/ncomms16078)
Supplement: Supplementary Information [file ncomms16078-s1.pdf]

File Name: Supplementary Information
Description: Supplementary Figures

File Name: Supplementary Data 1
Description: List of the FDA-approved anti-cancer agents used for HTS.

File Name: Supplementary Data 2
Description: List of the pathway inhibitor library used for HTS.

File Name: Supplementary Data 3
Description: Summary of siRNA oligos and primers for qRT-PCR.

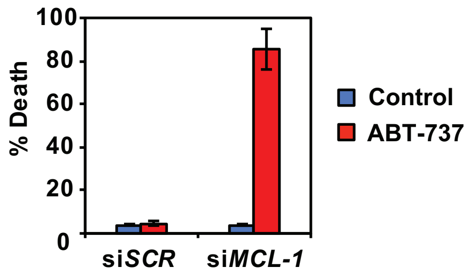

Supplementary Figure 1 | Knockdown of *MCL-1* sensitizes H196 cells to ABT-737. H196 cells, transfected with the scrambled siRNA (si*SCR*) or siRNA against *MCL-1*, were treated with vehicle or ABT-737 for 24 hr. Cell death was quantified by annexin-V staining (mean ± s.d., n = 3 independent experiments).

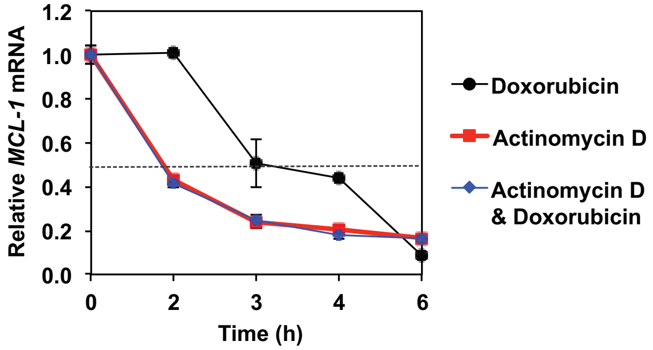

Supplementary Figure 2 | The mRNA levels of *MCL-1* in H196 cells treated with the indicated agents (2 μM) for the indicated times were assessed by qRT-PCR. Data were normalized against β-Actin (mean ± s.d., n = 2 independent experiments).

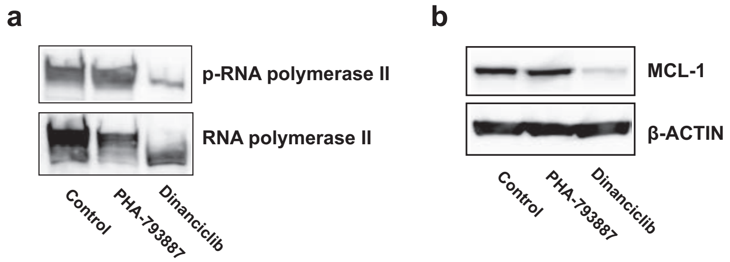

Supplementary Figure 3 | PHA-793887 neither suppresses the Ser2 phosphorylation of Pol II nor reduces MCL-1. (**a**) H196 cells, treated with vehicle or the indicated agents for 3 hr, were assessed by immunoblot analysis using the indicated antibodies. (**b**) H196 cells, treated with vehicle or the indicated agents for 6 hr, were assessed by immunoblot analysis using the indicated antibodies.

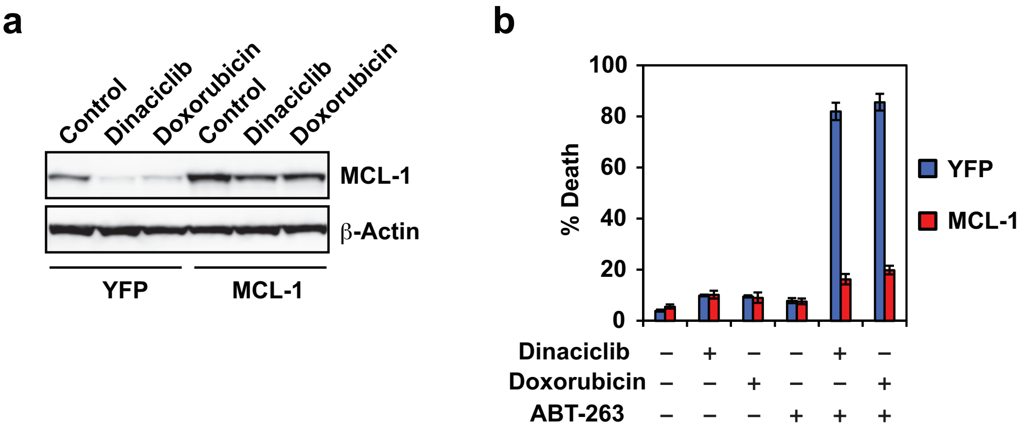

Supplementary Figure 4 | Overexpression of MCL-1 protects H196 cells from apoptosis triggered by the combination of ABT-263 and dinaciclib or doxorubicin. (**a**) H196 cells infected with lentivirus expressing YFP or MCL-1-IRES-YFP were treated the indicated agents for 6 hr and subsequently assessed by immunoblot analysis using the indicated antibodies. (**b**) H196 cells expressing YFP or MCL-1-IRES-YFP were treated the indicated agents for 24 hr. Cell death was quantified by annexin-V staining (mean ± s.d., n = 3 independent experiments).

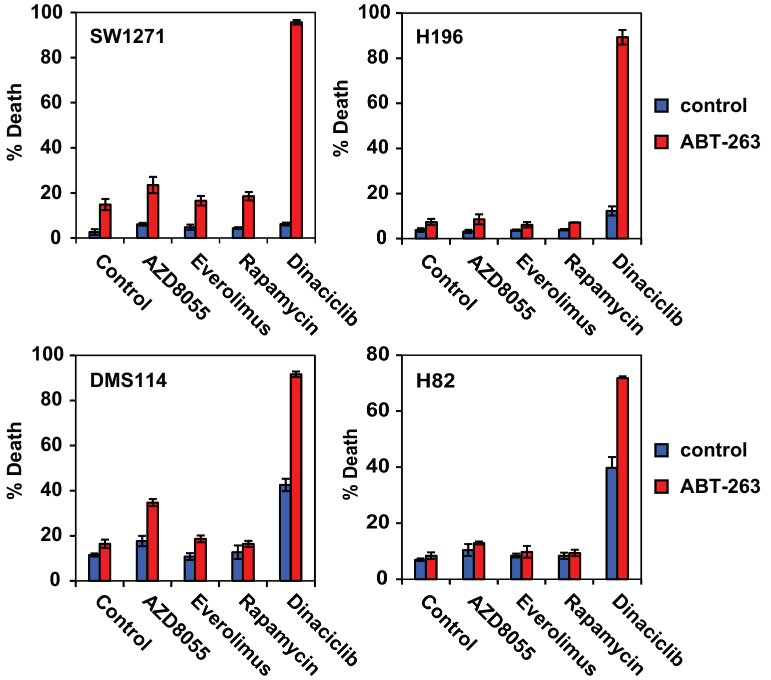

Supplementary Figure 5 | The combination of ABT-263 and dinaciclib is more potent in triggering apoptosis in SCLC than the combination of ABT-263 and mTOR inhibitors. The indicated SCLC cell lines were treated with indicated agents for 24 hr. Cell death was quantified by annexin-V staining (mean ± s.d., n = 3 independent experiments).

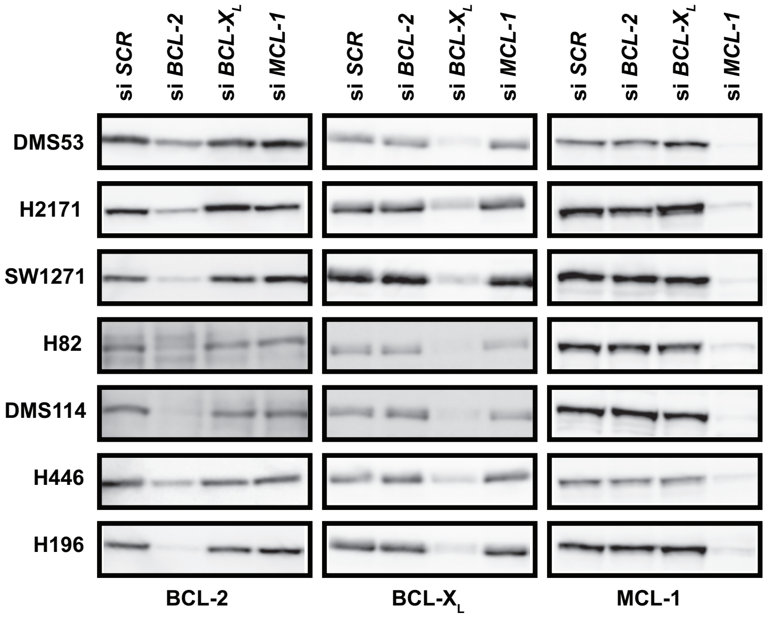

Supplementary Figure 6 | Confirmation of siRNA-mediated knockdown by immunoblot analysis. The indicated SCLC cell lines, treated with the indicated siRNA, were subject to immunoblot analysis using the indicated antibodies.

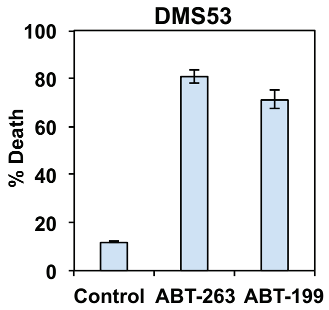

Supplementary Figure 7 | Both ABT-263 and ABT-199 induce robust apoptosis in DMS53 cells. DMS53 cells were treated with vehicle, ABT-263 (1μM), or ABT-199 (1μM) for 24 hr. Cell death was quantified by annexin-V staining (mean ± s.d., n = 3 independent experiments).

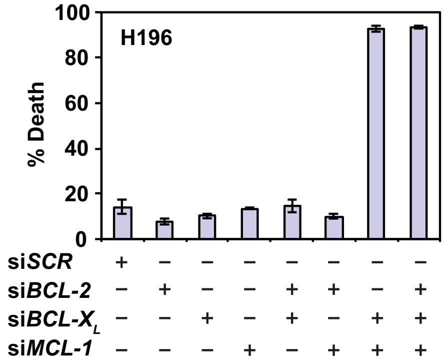

Supplementary Figure 8 | H196 is not addicted to any single anti-apoptotic BCL-2 member for survival. H196 cells were transfected with the indicated siRNA. Cell death was quantified by annexin-V staining (mean ± s.d., n = 3 independent experiments).

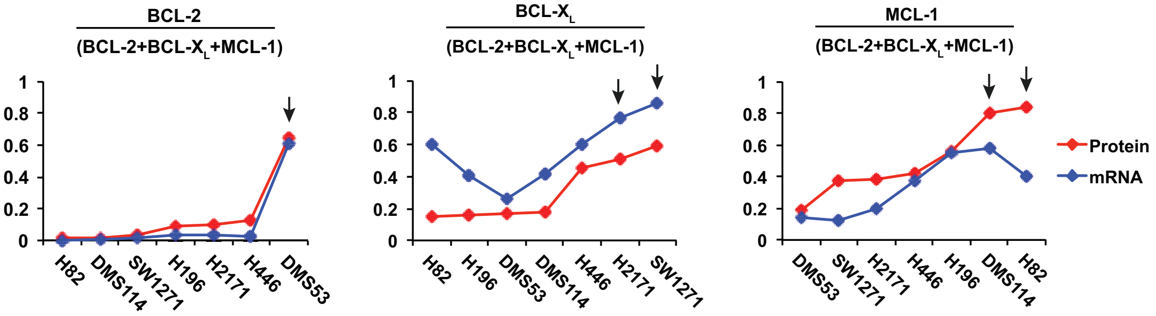

Supplementary Figure 9 | The protein ratios are superior to the mRNA ratios in predicting the addiction of individual anti-apoptotic BCL-2 members in SCLC. The protein or mRNA expression ratios of BCL-2, BCL-X_L, or MCL-1 to combined BCL-2, BCL-X_L, and MCL-1 were determined in the indicated SCLC cell lines based on two representative immunoblot or quantitative RT-PCR analyses. Arrows indicate the cell lines that display addiction to BCL-2, BCL-X_L, or MCL-1 for survival.

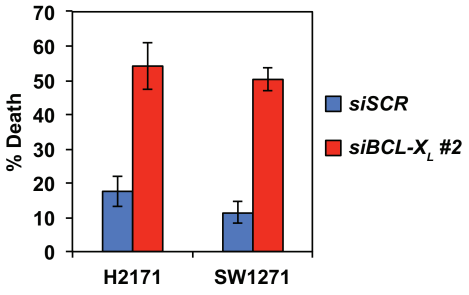

Supplementary Figure 10 | Knockdown of *BCL-X_L* induces apoptosis in H2171 and SW1271 cells. H2171 and SW1271 cells were transfected with the scrambled siRNA (si*SCR*) or siRNA against *BCL-X_L*. Cell death was quantified by annexin-V staining (mean ± s.d., n = 3 independent experiments).

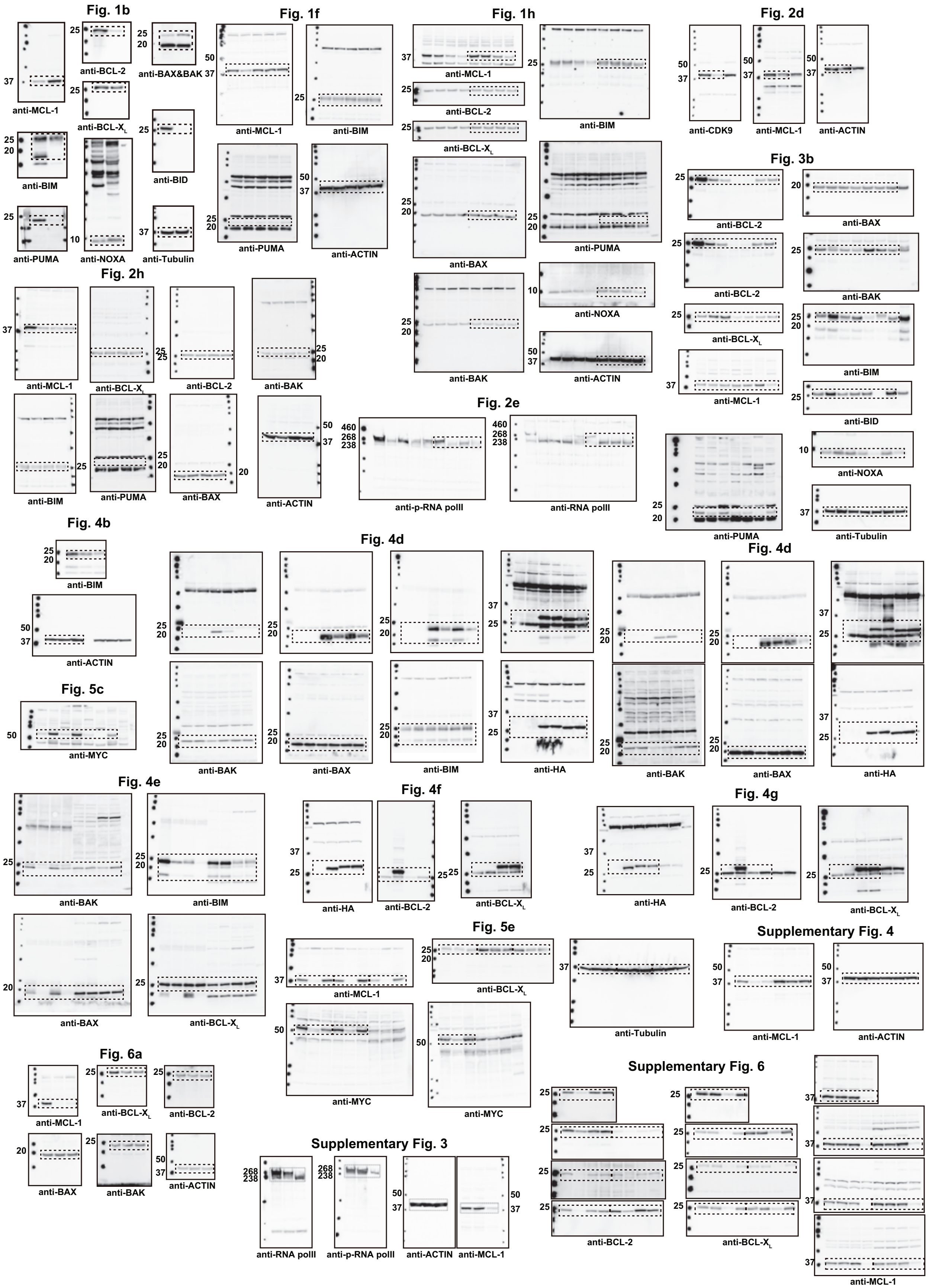

Fig. 1b
anti-MCL-1
anti-BCL-2
anti-BAX&BAK
anti-BCL-XL
anti-BIM
anti-BID
anti-PUMA
anti-NOXA
anti-Tubulin
Fig. 1f
anti-MCL-1
anti-BIM
anti-PUMA
anti-ACTIN
Fig. 1h
anti-MCL-1
anti-BCL-2
anti-BCL-XL
anti-BIM
anti-BAX
anti-PUMA
anti-BAK
anti-NOXA
anti-ACTIN
Fig. 2d
anti-CDK9
anti-MCL-1
anti-ACTIN
Fig. 3b
anti-BCL-2
anti-BAX
anti-BCL-2
anti-BAK
anti-BCL-XL
anti-BIM
anti-MCL-1
anti-BID
anti-NOXA
anti-PUMA
anti-Tubulin
Fig. 2h
anti-MCL-1
anti-BCL-XL
anti-BCL-2
anti-BAK
anti-BIM
anti-PUMA
anti-BAX
anti-ACTIN
Fig. 2e
anti-p-RNA polII
anti-RNA polII
Fig. 4b
anti-BIM
anti-ACTIN
Fig. 4d
anti-BAK
anti-BAX
anti-BIM
anti-HA
Fig. 4d
anti-BAK
anti-BAX
anti-HA
Fig. 5c
anti-MYC
Fig. 4e
anti-BAK
anti-BIM
anti-BAX
anti-BCL-XL
Fig. 4f
anti-HA
anti-BCL-2
anti-BCL-XL
Fig. 4g
anti-HA
anti-BCL-2
anti-BCL-XL
Fig. 5e
anti-MCL-1
anti-BCL-XL
anti-Tubulin
anti-MYC
anti-MYC
Supplementary Fig. 4
anti-MCL-1
anti-ACTIN
Fig. 6a
anti-MCL-1
anti-BCL-XL
anti-BCL-2
anti-BAX
anti-BAK
anti-ACTIN
Supplementary Fig. 3
anti-RNA polII
anti-p-RNA polII
anti-ACTIN
anti-MCL-1
Supplementary Fig. 6
anti-BCL-2
anti-BCL-XL
anti-MCL-1

Supplementary Figure 11 | Full scans of immunoblots. In some experiments, membranes were cut prior to probing each strip with a separate antibody.
